# P-2363. Efficacy Of SARS–CoV-2 Specific Antiviral Therapy for Enteroviral Myalgic Encephalomyelitis/ChronicFatigue Syndrome

**DOI:** 10.1093/ofid/ofae631.2514

**Published:** 2025-01-29

**Authors:** John K Chia, David Wang

**Affiliations:** EV Med Research, Lomita, California; EV Med Research, Lomita, California

## Abstract

**Background:**

Etiology remains elusive for Myalgic Encephalomyelitis/Chronic Fatigue Syndrome (ME/CFS), and no treatment exists. Antivirals had no efficacy in randomized clinical trials (RCT) for Epstein-Barr Virus and HHV-6. Enteroviruses (EV) have been implicated, but no antivirals are available. Many patients who received SARS–CoV-2-specific antiviral drugs for acute Covid-19 (COV19) infection experienced significant improvement of prior ME/CFS symptoms. This study summarizes their responses to antivirals for SARS-Cov-2.

**Methods:**

Neutralizing Antibody (NA) for Coxsackievirus B (CVB)1-6 and Echovirus 6, 7, 9, 11, 30 were done by ARUP lab. Enterovirus Protein (EVP) of Peripheral Blood Leukocytes (PBL) was determined by Western Blot. ME/CFS patients fulfilled Canadian consensus criteria, and had either elevated NA for enteroviruses and/or positive EVP in PBL. ME/CFS patients hospitalized for acute COV19 infection and patients without COV19, were given 5-10 days of IV Remdesivir (Rem) +/- immune modulators. Controls: 20 ME/CFS patients seen concurrently without Remdesivir treatment. Other ME/CFS patients (non-COV19) were given Nirmaltrelvir/Ritonavir (PAX) daily for 10 days +/- one repeat.

The energy index (EI) was monitored by the patients before, during and after treatment. Significant improvement was defined as > 30%.

**Results:**

15/20 (75%) ME/CFS patients – 10/12 hospitalized, 5/8 non-COV19 patients – responded to IV Rem 2-6 weeks after infusions; remission lasted 6-8 weeks to 6-9 months before relapse. Of Controls: 2/20 had mild improvement (< 0.001, X 2 ).

104/200 (52%) of PAX-treated ME/CFS patients improved, often within 2-3 days; all relapsed within days to weeks after treatment. 66%, 33% and 44% of CVB4+, CVB3+, non-CVB3,4+ patients responded to treatment, respectively. EVP decreased and increased with clinical response and relapse.

**Conclusion:**

Rem and PAX demonstrated clinical efficacy in ME/CFS patients with chronic enterovirus infections. Placebo-controlled RCT will be needed to clarify the role of antivirals in ME/CFS.

**Disclosures:**

All Authors: No reported disclosures

